# Dietary factors that affect the risk of pre-eclampsia

**DOI:** 10.1136/bmjnph-2021-000399

**Published:** 2022-06-06

**Authors:** Abigail Perry, Anna Stephanou, Margaret P Rayman

**Affiliations:** Department of Nutritional Sciences, Faculty of Health and Medical Sciences, University of Surrey, Guildford, UK

**Keywords:** blood pressure lowering, dietary patterns, nutrient deficiencies, nutritional treatment, weight management

## Abstract

Pre-eclampsia affects 3%–5% of pregnant women worldwide and is associated with a range of adverse maternal and fetal outcomes, including maternal and/or fetal death. It particularly affects those with chronic hypertension, pregestational diabetes mellitus or a family history of pre-eclampsia. Other than early delivery of the fetus, there is no cure for pre-eclampsia. Since diet or dietary supplements may affect the risk, we have carried out an up-to-date, narrative literature review to assess the relationship between nutrition and pre-eclampsia. Several nutrients and dietary factors previously believed to be implicated in the risk of pre-eclampsia have now been shown to have no effect on risk; these include vitamins C and E, magnesium, salt, ω-3 long-chain polyunsaturated fatty acids (fish oils) and zinc. Body mass index is proportionally correlated with pre-eclampsia risk, therefore women should aim for a healthy pre-pregnancy body weight and avoid excessive gestational and interpregnancy weight gain. The association between the risk and progression of the pathophysiology of pre-eclampsia may explain the apparent benefit of dietary modifications resulting from increased consumption of fruits and vegetables (≥400 g/day), plant-based foods and vegetable oils and a limited intake of foods high in fat, sugar and salt. Consuming a high-fibre diet (25–30 g/day) may attenuate dyslipidaemia and reduce blood pressure and inflammation. Other key nutrients that may mitigate the risk include increased calcium intake, a daily multivitamin/mineral supplement and an adequate vitamin D status. For those with a low selenium intake (such as those living in Europe), fish/seafood intake could be increased to improve selenium intake or selenium could be supplemented in the recommended multivitamin/mineral supplement. Milk-based probiotics have also been found to be beneficial in pregnant women at risk. Our recommendations are summarised in a table of guidance for women at particular risk of developing pre-eclampsia.

## Introduction

Pre-eclampsia is a multisystem syndrome of pregnancy. It is one of the leading causes of maternal and perinatal morbidity and mortality.^1^ Pre-eclampsia complicates about 3%–5% of all pregnancies and is estimated to cause at least 42 000 maternal deaths annually.^2^ The International Society for the Study of Hypertension in Pregnancy (ISSHP) defines pre-eclampsia as de novo hypertension present after 20 weeks of gestation combined with proteinuria (>300 mg/day) or other maternal organ dysfunction, comprising renal insufficiency, liver involvement, neurological or haematological complications, uteroplacental dysfunction or fetal growth restriction.^3^

Pre-eclampsia results from placental malperfusion followed by syncytiotrophoblast stress that releases soluble factors into the circulation causing an early imbalance between proangiogenic and antiangiogenic factors.^4^ Pathways that influence the development of pre-eclampsia include genetic, epigenetic, lifestyle and environmental factors.^4^ However, there is little published information on diet and pre-eclampsia. In this narrative review, we will concentrate on relevant dietary components that interact with lifestyle and environment. We will include factors that affect metabolic function, including weight, weight gain, hypertension, adverse lipid profile, inflammation, dietary patterns and factors that provide endothelial protection.^5–9^ Our investigation will develop a set of nutritional guidelines to reduce the risk of pre-eclampsia in pregnancy, this being of particular importance for those at high risk. We hypothesise that modification of diet and nutritional intake may reduce an individual’s risk of pre-eclampsia.

## Materials and methods

### Search strategy

Following a brief review of the literature on diet/nutrients and pre-eclampsia, we decided which diet/nutrients/nutritional supplements to search for in this review. The key factors of interest were maternal weight before and during pregnancy, calcium, vitamin D, selenium, multivitamins/multiminerals, fibre, prebiotics and probiotics and dietary patterns. Dietary factors that were also considered despite a previous lack of evidence included antioxidants, magnesium, salt, ω-3 long-chain polyunsaturated fatty acids (LC-PUFAs), zinc, iodine and folate/folic acid. The PubMed database was searched for articles between January 2000 and November 2021 on the effect of various nutrients/nutritional supplements, dietary factors and maternal weight on the risk of pre-eclampsia using key search terms (see table 1).

**Table 1:** Search terms used for article selection

Factor	Key search terms
Antioxidants	preeclampsia AND antioxidants
Calcium	preeclampsia AND calcium
Dietary pattern	preeclampsia AND ‘dietary pattern’
Fibre	preeclampsia AND fibre OR fiber
Folate/folic acid	preeclampsia AND folate OR preeclampsia AND ‘folic acid’
Iodine	preeclampsia AND iodine
Magnesium	preeclampsia AND magnesium
Maternal weight	preeclampsia AND ‘gestational weight gain’ OR preeclampsia AND ‘maternal BMI’
Multivitamins/multiminerals	preeclampsia AND multivitamin OR preeclampsia AND multimineral
Omega-3 long-chain polyunsaturated fatty acids	preeclampsia AND omega-3 long-chain polyunsaturated fatty acids
Salt	preeclampsia AND salt
Selenium	preeclampsia AND selenium
Vitamin B_12_	preeclampsia AND ‘vitamin B12’
Vitamin D	preeclampsia AND ‘vitamin D’
Zinc	preeclampsia AND zinc

### Inclusion and exclusion criteria

Abstracts and finally articles were reviewed to determine their eligibility for inclusion. They were eligible if they assessed the risk of pre-eclampsia as defined by the ISSHP. Inclusion also required that the article investigated at least one nutrient and its effect on the risk of pre-eclampsia.

### Data extraction

Reference lists from selected articles were manually checked to identify further relevant studies. Two hundred and ninety papers were used to compile the review of which 169 have been cited. These included data from randomised control, observational and case–control studies, articles on the potential protective mechanism of the action of nutrients and dietary factors and published nutritional guidelines on their intake and status.

## Results and discussion

### Previously investigated foods and nutrients with no or low evidence of benefit

From the latter part of the last century, a range of dietary components was suggested as having the potential to reduce the risk of pre-eclampsia. Since that time, some of those previously identified factors have now been shown to have no effect on risk. Further information on those factors is included in the online supplemental information entitled *Foods and nutrients with low or no evidence of benefit* and a summary of the evidence for each nutrient and its lack of efficacy is shown in table 2.

10.1136/bmjnph-2021-000399.supp1 Supplementary data

**Table 2:** Evidence from different studies on nutrients that show a lack of benefit on pre-eclampsia risk or prevention

Nutrient and citation	Type of study	Participants (n)	Main outcomes	Findings	Conclusion
**Vitamin C/vitamin E**					
Poston *et al* 156	RCT with 1000 mg C and 400 IU E *or* placebo per day from the 2nd trimester until delivery	2404 UK women at increased risk of PE	PE, low birth weight, small size for gestational age	Women treated with vitamins C and E did not have a reduced risk; RR 0.97, 95% CI 0.80 to 1.17. No difference was found in severe PE between the groups; RR 1.17, 95% CI 0.8 to 1.68.	Vitamin C and E supplementation in the 2nd trimester did not reduce the risk of PE in women at risk.
Klemmensen *et al* 157	Prospective cohort study of vitamin intake	n=49 373 Danish women	Vitamin intake from diet and supplement from previous 4 weeks estimated from 25-week FFQ. PE, eclampsia, HELLP.	Vitamin C was not associated with an increased risk of any type of PE. For severe PE/eclampsia/HELLP, there was a decreasing trend with increasing dietary vitamin C intake (p=0.01). Vitamin E was associated with an increased risk of all types of PE; OR 1.19, 95% CI 1.00 to 1.42, and an increased risk of severe PE/eclampsia/HELLP; OR 1.46, 95% CI 1.02 to 2.09.	Vitamin C showed an increased incidence of severe pre-eclampsia, eclampsia and HELLP. Vitamin E increased the risk of disease.
Rumbold *et al* 158	Cochrane Database of Systematic Reviews/meta-analysis	10 eligible studies n=6533	PE, severe PE (HELLP), preterm birth, SGA, baby death	No significant difference between antioxidant and control groups in risk of PE (RR 0.73, 95% CI 0.51 to 1.06) and severe PE (RR 1.25, 95% CI 0.89 to 1.76).	No difference in PE risk. Women reported abdominal pain, required antihypertensive therapy and hospital admission.
Basaran *et al* 159	Systematic review/meta-analysis of the effectiveness of combined vitamin C/E versus placebo supplement	9 studies, n=9833 in vitamin C/E group n=9842 in placebo group	PE, gestational hypertension, placental abruption	PE incidence in vitamin C/E was 9.7% (949/9833) and 9.5% (946/9842) in placebo group. No difference in RR of PE found between vitamin C/E and placebo group; RR 0.98, 95% CI 0.87 to 1.10.	Vitamin C/E supplementation does not reduce the risk of PE and should not be recommended to prevent PE.
Salles *et al* 160	Systematic review of RCTs that evaluated the use of antioxidants versus placebo	15 studies with 21 012 women and 21 647 fetuses	PE	No significant difference in PE incidence was observed; RR 0.92, 95% CI 0.82 to 1.04. Side effects were numerically but non-significantly more frequent in the antioxidant group compared with the placebo group.	Prevention of PE and other outcomes was not observed.
**Fish oils or ω-3 long-chain polyunsaturated fatty acids (LC-PUFA)**					
Williams *et al* 161	Case–control study	22 PE women, 40 control women	Polyunsaturated fatty acids in women’s erythrocytes were measured.	Women with the lowest levels of ω-3 fatty acids were 7.6 times more likely to have had their pregnancies complicated by pre-eclampsia than women with the highest levels of ω-3 fatty acids (95% CI 1.4 to 40.6).	Low levels of ω-3 fatty acids and high levels of some ω-6 fatty acids, particularly AA, were associated with an increased PE risk.
Oken *et al* 46	Prospective cohort study of intake	n=1718 women	First-trimester intake of Ca, ω-3/ω-6/trans fatty acids, folate, Mg, vitamins C, D, E with PE/PIH	A somewhat lower risk of PE was associated with higher intake of the elongated ω-3 fatty acids DHA and EPA (OR 0.84, 95% CI 0.69 to 1.03 per 100 mg/day). There were no effects of other nutrients on PE.	There was a *potential* benefit of ω-3 fatty acids in PE prevention.
Szajewska *et al* 162	Systematic review of RCTs comparing ω-3 LC-PUFA supplementation with placebo/no supplementation	6 eligible studies	PE, eclampsia, pregnancy duration, preterm delivery, low birth weight	No significant difference was observed in PE rate between supplemented group and placebo group; RR 0.73, 95% CI 0.22 to 2.37. ω-3 supplementation was associated with a higher duration of pregnancy.	ω-3 supplementation increases the duration of pregnancy and head circumference but does not decrease the rate of PE.
Makrides *et al* 163	Systematic review of RCTs of supplement with fish oil/other prostaglandin precursor with placebo/no treatment	6 eligible studies n=2783 women	PE, preterm birth, low birth weight, SGA	No difference on the risk of PE between supplemented and placebo groups.	There is not enough evidence to support the use of fish oils or prostaglandins on the risk of PE.
Horvath *et al* 164	RCT meta-analysis comparing ω-3 LC-PUFA supplement with placebo or no supplementation	4 studies; 1264 women with high-risk pregnancies	Duration of pregnancy, birth weight, PIH, PE	The rate of PE was similar in both groups. Supplementation was associated with a significantly lower rate of early preterm delivery.	There is no evidence to support the use of ω-3 LC-PUFA to reduce the rate of PE.
Imhoff-Kunsch *et al* 165	Systematic review of RCTs where ω-3 LC-PUFAs were provided to pregnant women for ≤1 trimester	15 eligible studies	PE, maternal BP, gestational duration, preterm birth	ω-3 LC-PUFA supplementation was not associated with maternal blood pressure, infant death, stillbirth or PE risk. However, women receiving ω-3 LC-PUFA had a 26% lower risk of early preterm delivery.	There was no benefit of the use of ω-3 LC-PUFA on the risk of PE.
**Low-salt diet**					
Duley *et al* 166	Systematic review	2 studies, 603 women	PE	No difference was observed between dietary salt restriction and a normal diet on the risk of pre-eclampsia; OR 1.11, 95% CI 0.46 to 2.66.	Evidence is low to assess whether advice to restrict salt during pregnancy is beneficial.
**Magnesium (Mg) supplementation**					
Makrides *et al* 167	Systematic review of RCTs and quasi-RCTs that assessed the effects of dietary Mg supplement	10 eligible studies in 9090 women	Perinatal mortality, SGA, maternal mortality, PE	There was no association between magnesium supplementation and the risk of perinatal mortality or small for gestational age. There was no effect on PE (RR 0.87, 95% CI 0.58 to 1.32; three trials, 1042 women).	The evidence is of low quality to show that magnesium supplementation can be beneficial during pregnancy.
de Araújo *et al* 168	RCT. 300 magnesium citrate/placebo per day given from 12 to 20 weeks until delivery.	n=318 women	PE	18.1% of women in magnesium group and 19.7% of women in the control group developed PE; OR 0.90, 95% CI 0.48 to 1.69.	Magnesium supplementation does not reduce the risk of PE.
**Zinc supplementation**					
Zahiri Sorouri *et al* 169	RCT with 400 µg folic acid and 30 mg ferrous sulfate, with/without 15 mg zinc sulfate per day from the 16th gestational week	n=540	PE, low birth weight, macrosomia, head circumference, length, preterm delivery, gestation at birth	No significant differences were found between the zinc-supplemented group and the no-zinc group on the risk of PE.	Zinc supplementation at 15 mg/day does not improve pregnancy outcomes.

AA, arachidonic acid; BP, blood pressure; DHA, docosahexaenoic acid; EPA, eicosapentaenoic acid; FFQ, Food Frequency Questionnaire; HELLP, haemolysis-elevated liver enzymes and low platelet count; PE, pre-eclampsia; PIH, pregnancy-induced hypertension; RCT, randomised controlled trial; RR, risk ratio; SGA, small for gestational age.

### Obesity and gestational weight gain

#### Consequences of obesity in pre-eclampsia

The clearest link between diet and pre-eclampsia is the effect of weight and gestational weight gain (GWG) on risk. Obesity is a growing threat to pregnant women with 20%–40% of women in Europe and USA gaining more weight than recommended during pregnancy.^10^ Obesity increases oxidative stress, stimulates an inflammatory response and damages vascular endothelial cells, factors associated with pre-eclampsia.^11^ Obesity and pre-eclampsia share common characteristics such as hyperinsulinaemia, insulin resistance, endothelial dysfunction and an inflammatory environment characterised by increased leptin and reduced adiponectin levels.^12–14^ Leptin specifically reduces the proliferation of cytotrophoblasts and increases blood pressure (BP) and placental growth factor.^8 15 16^

#### Evidence for a link between pre-pregnancy body mass index and pre-eclampsia

**Figure 1:**
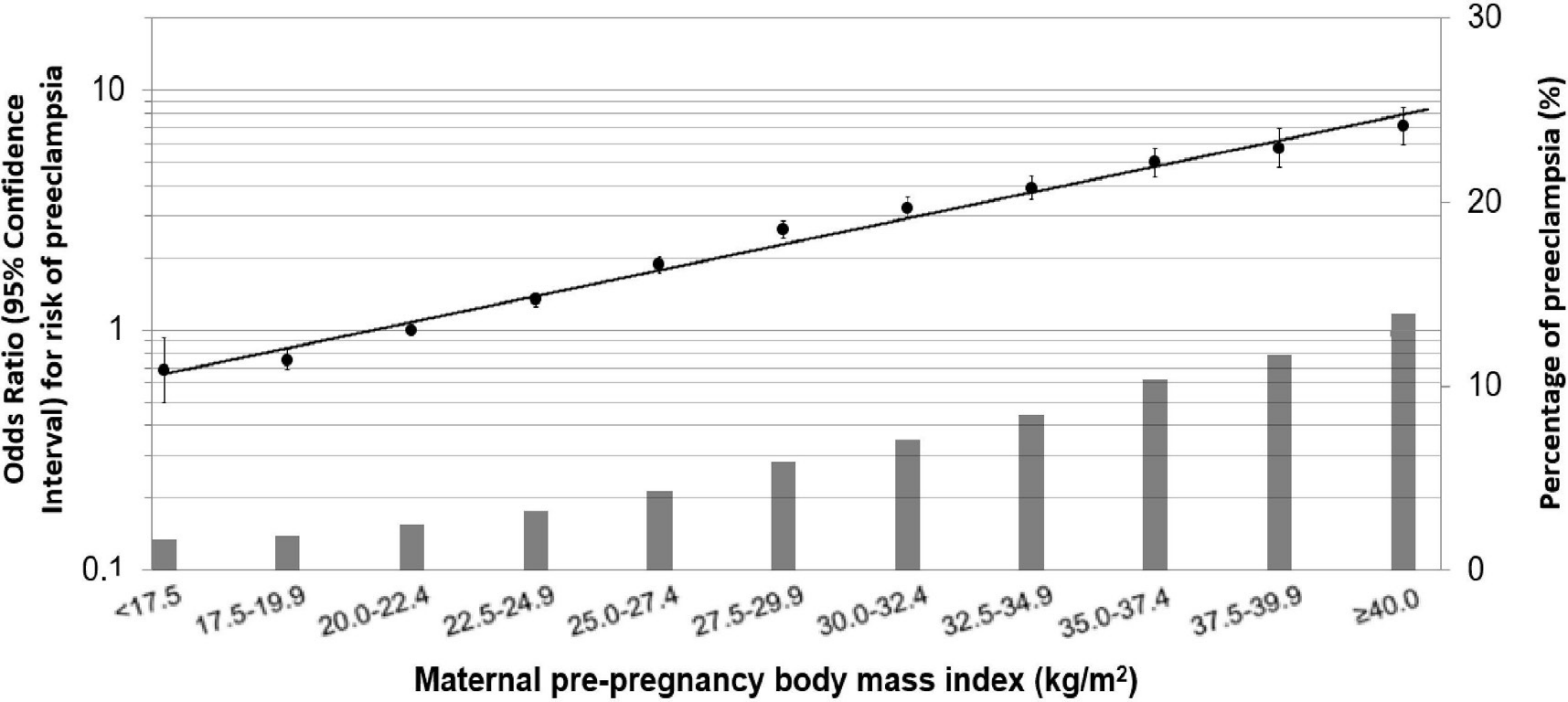
The OR for pre-eclampsia and the percentage of women with pre-eclampsia against maternal pre-pregnancy body mass index (BMI).^8^ Values are ORs (95% CIs) on a log scale from multilevel binary logistic regression models that reflect the risk of pregnancy complications per pre-pregnancy BMI group compared with the reference group (largest group, 20.0–22.4 kg/m^2^). The bars represent the percentage of pre-eclampsia per BMI group. Models are adjusted for maternal age, educational level, parity and smoking habits during pregnancy^8^ (adapted from Santos *et al* 8).

Pre-pregnancy body mass index (BMI) is positively associated with the risk of pre-eclampsia. A 2015 population-based cohort study in British Columbia including 226 958 singleton pregnancies showed that a 10% difference in pre-pregnancy BMI was associated with a 10% reduction in pre-eclampsia risk.^17^ Having adjusted for fat mass, the *Norwegian Fit for Delivery* study observed an independent association between pre-pregnancy BMI and pre-eclampsia, for example, in week 36, OR 1.71, 95% CI 1.26 to 2.31, p=0.0010.^11^ A meta-analysis of 39 European, North American and Australian cohorts showed that pre-pregnancy obesity grade 3 (BMI >40 kg/m^2^) increases the risk of any pregnancy complication, for example, 34.6% of mothers developed pre-eclampsia (OR 6.50, 95% CI 5.48 to 7.73).^8^ Figure 1 illustrates the risk of pregnancy complications with BMI status observed in this study.^8^

#### Evidence for a link between GWG and pre-eclampsia

In a population-based retrospective study in Missouri, results showed that women who were superobese with a high rate of GWG were at an increased risk of pre-eclampsia than were normal-weight women (OR 7.52, 95% CI 2.70 to 21.0).^18^ The *Norwegian Fit for Delivery* study examined GWG in 550 pregnant women and explored possible associations with body composition.^11^ Women who developed pre-eclampsia gained more weight than women who did not (difference 3.7 kg, p=0.004), with a 3.5 kg difference in total body water observed in week 36. A 1 kg increase in GWG was associated with an adjusted 1.3 times higher odds of pre-eclampsia (OR 1.31, 95% CI 1.15 to 1.49, p<0.001).^11^ Independently, fat mass in week 36 was *inversely* associated with pre-eclampsia (OR 0.79, 95% CI 0.68 to 0.92). The difference found in total body water suggests that oedema drives the association between GWG (which is more strongly associated with late-onset pre-eclampsia) and pre-eclampsia.^11 19^ The authors question whether excessive GWG is a causal factor in the pathophysiology of pre-eclampsia, or rather an indication of early endothelial dysfunction leading to excess fluid retention from an early stage of pregnancy.^11^ As far as hypertensive normal-weight and overweight women are concerned, a recent cohort study showed that high GWG increases the risk of pre-eclampsia by 40%–90%.^20^

#### Weight management interventions in pre-eclampsia

Although weight loss is not recommended during pregnancy, it may be beneficial for reducing pre-eclampsia risk.^21^ A systematic review and meta-analysis published in 2012 that included 44 randomised controlled trials (RCTs) assessed three interventions for the risk of pre-eclampsia.^21^ Around 7278 women were randomised into (1) diet, (2) physical activity, or (3) a mixed approach of diet and physical activity interventions.^21^ A reduction in pre-eclampsia by 26% was observed as an overall effect from the three interventions.^21^ Moreover, reduction in pre-eclampsia risk by 33% and the largest reductions in GWG were observed in the dietary intervention group (risk ratio (RR) 3.84 kg, 95% CI 2.45 to 5.22) with improved pregnancy outcomes compared with other interventions, though substantial heterogeneity in maternal weight gain was observed (I^2^=92%).^21^ However, the most recent systematic review with data collected through February 2021 incorporated 68 studies (n=25 789) and found no significant association between GWG interventions (diet, exercise and/or behavioural counselling) and risk of pre-eclampsia.^22^ A similar outcome was found in a patient data meta-analysis of diet and lifestyle interventions in 9320 women; GWG was reduced by an average of 0.70 kg but there was no effect on pre-eclampsia risk.^23^ However, if excessive GWG is a consequence of pre-eclampsia rather than a causal factor, a focus on diet quality or other health behaviours that may modify the stress response to pregnancy may be more fruitful.^17^

#### Recommendation

The Institute of Medicine defines recommended GWG for pregnant women, stratified by pre-pregnancy BMI (see table 3).^24^ Interpregnancy weight gain has also been shown to increase the risk of pre-eclampsia and of recurrent pre-eclampsia; therefore, excessive weight gain during pregnancy and between pregnancies should be avoided.^20 25 26^ The aim is to maintain a healthy weight prior to conception. However, in women at risk of pre-eclampsia who are overweight and/or obese, dietary interventions to reduce excessive GWG may be beneficial both for the mother and the baby. Attempting to lose weight during pregnancy is generally not advised and may lead to adverse outcomes such as fetal growth restriction.^27^

**Table 3:** Recommendations for total weight gain during pregnancy, by pre-pregnancy BMI^24^

Pre-pregnancy BMI (kg/m^2^)	Recommended total weight gain (kg)
Underweight <18.5	12.5–18
Healthy weight 18.5–24.9	11.5–16
Overweight 25.0–29.9	7–11.5
Obese ≥30	5–9

BMI, body mass index.

### Fibre

#### Protective mechanisms by which fibre acts in pre-eclampsia

A higher intake of dietary fibre aids in weight maintenance and is associated with a reduced risk of cardiovascular disease.^28 29^ In comparison to normotensive women, pre-eclamptic women have significantly higher serum triglycerides and low-density lipoprotein (LDL) cholesterol, known to increase the risk of hypertension and cardiovascular disease.^7^ In a prospective cohort study from Washington State, plasma lipid concentrations were measured in maternal blood samples at 13 weeks’ gestation from 57 women who developed pre-eclampsia and 510 who remained normotensive and served as controls.^6^ Women who subsequently developed pre-eclampsia had 10.4%, 13.6% and 15.5% higher concentrations of LDL cholesterol, triglycerides and LDL/high-density lipoprotein (HDL) ratios, respectively, than did control subjects (p<0.05).^6^ Dietary fibre appears to be capable of attenuating such dyslipidaemia,^30–32^ while reducing BP and inflammation, a key feature of pre-eclampsia.^33–36^

#### Evidence from epidemiological studies for fibre intake reducing pre-eclampsia risk

**Figure 2:**
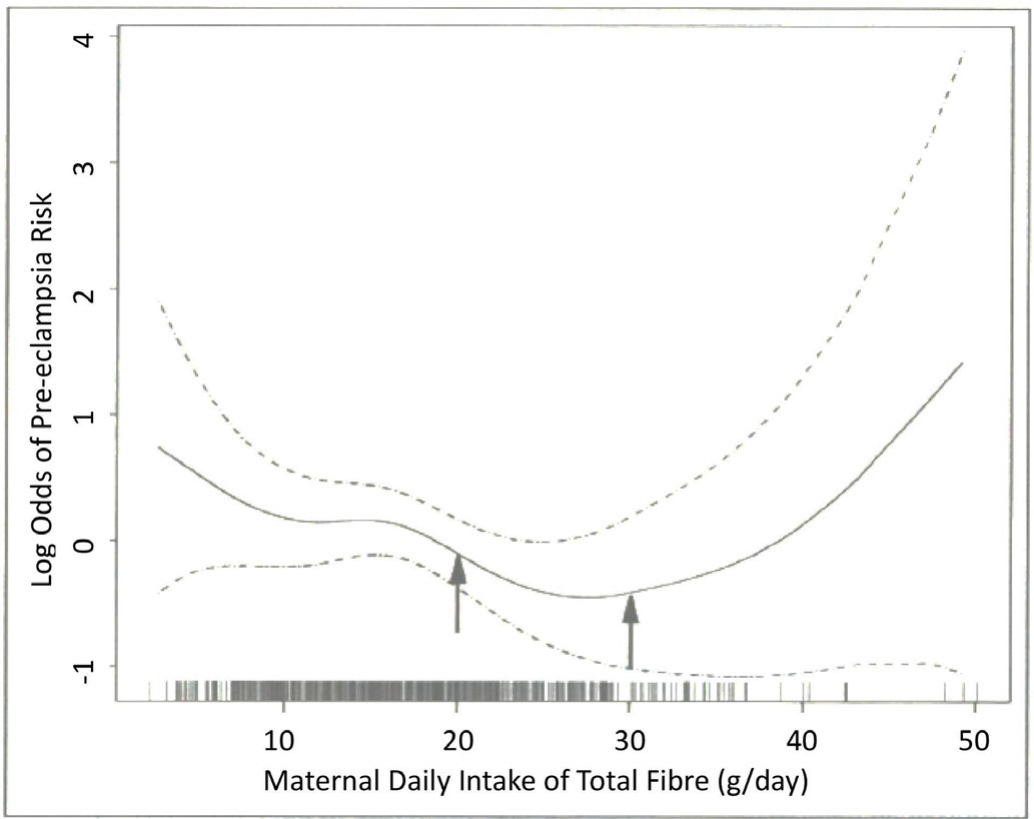
Relationship between total dietary fibre intake (g/day) and the risk of pre-eclampsia (solid line), with 95% CI (dotted lines). Arrows indicate previous Recommended Dietary Allowance of 20–30 g/day of total fibre for pregnant women in the USA. The vertical bars along the dietary fibre axis indicate the density of the data (adapted from Frederick *et al* 37).

A 2005 case–control study assessed fibre intake in 172 women with pre-eclampsia and 339 controls using Food Frequency Questionnaires (FFQs).^37^ After adjusting for confounders, women in the highest quartile of fibre intake (>24.3 g/day) had a 51% reduced risk of developing pre-eclampsia (OR 0.49, 95% CI 0.24 to 1.00) than women in the lowest quartile of fibre intake (<13.1 g/day).^37^ Moreover, use of logistic regression procedures suggested that as total fibre intake increased to around 27–30 g/day, pre-eclampsia risk decreased. However, above intakes of 30 g of fibre per day, risk of pre-eclampsia increases (see figure 2).^37^

A 2008 study investigated the fibre intake of 1538 pregnant women living in Washington, referred to above.^6 32^ FFQs were used to assess dietary fibre intake 3 months prior to and during gestation. After adjusting for confounders, the RR of pre-eclampsia for women in the highest (≥21.2 g/day) versus the lowest quartile (<11.9 g/day) of fibre intake was 0.28 (95% CI 0.11 to 0.75). Mean triglyceride concentrations were lower (−11.9 mg/dL, p=0.02) and HDL cholesterol concentrations were higher (+2.63 mg/dL, p=0.09) for women in the highest quartile versus those in the lowest quartile, showing that dietary fibre intake may attenuate pregnancy-associated dyslipidaemia.^32^

#### Recommendation

A high-fibre diet is recommended for pregnant women. They should aim for a dietary fibre intake of 25–30 g/day to reduce the risk of pre-eclampsia. Amounts of fibre above 30 g may even increase the risk of pre-eclampsia.^38^

### Probiotics and prebiotics

Probiotics are live microorganisms, that is, bacteria and yeasts with several functions including restoring the gut microbiome, improving cholesterol and BP levels.^39^ Prebiotics are foods that are used by bacteria to stimulate the growth of the indigenous population of bifidobacteria to confer probiotic health benefits on the host.^40 41^ Probiotic food has been shown to help lower the risk of pre-eclampsia in pregnant women by reducing inflammation in the placental trophoblast cells, reducing BP, and systemic inflammation.^38 42^ Prenatal prebiotics significantly increased beneficial maternal intestinal *Bifidobacterium*.^42^

#### Observational studies of the effect of probiotics in pre-eclampsia

The Norwegian Mother and Child Cohort Study (MoBa) examined the association between milk-based probiotics in 33 399 pregnant women and pre-eclampsia development.^38^ An intake of about 140 mL/day of a probiotic product was associated with a reduced risk of all pre-eclampsia (OR 0.80, 95% CI 0.66 to 0.96) with a more notable effect in severe pre-eclampsia (OR 0.61, 95% CI 0.43 to 0.89).^38^ An RCT showed that pregnant women who consumed a probiotic yoghurt for 9 weeks had significantly reduced levels of high-sensitivity C-reactive protein than controls, suggesting that probiotics had a beneficial effect in reducing systemic inflammation.^43^

A prospective observational cohort study in Norway investigated the timing of probiotic intake before, during or late in pregnancy and its association with pre-eclampsia.^44^ Around 37 050 nulliparous women were included of whom 1851 were diagnosed with pre-eclampsia and 550 with severe pre-eclampsia.^44^ The results showed that consumption of probiotics significantly reduced pre-eclampsia risk when consumed during late pregnancy (OR 0.80, 95% CI 0.68 to 0.94); no such association was found when considering consumption of probiotics pre-pregnancy and during early pregnancy.^44^

#### Recommendation

A protective association was seen in the use of milk-based probiotic products on pre-eclampsia risk, including severe pre-eclampsia, especially when consumed during late pregnancy. Pregnant women should therefore aim to incorporate dairy-based probiotics into their diet. Further research is needed to determine quantity and timing.

### Dietary patterns

#### Importance of dietary patterns to the risk of pre-eclampsia

Dietary patterns before and during pregnancy may play a crucial role in the development of gestational hypertension, including pre-eclampsia.^45^ Although limited, there is some evidence to suggest that diets higher in fruits and vegetables, nuts, whole grains, legumes, fish and vegetable oils are protective against hypertensive disorders.^45^ Observational evidence for the effect of various dietary patterns on pre-eclampsia risk is outlined below.

#### Diets high in fruits and vegetables

Research suggests that diets characterised by higher intakes of fruits and/or vegetables reduce the risk of pre-eclampsia.^45–53^ For example, a Norwegian observational study assessed the diet of 23 000 mothers and found that a diet characterised by higher vegetables, plant foods and vegetable oils was associated with a reduced risk of pre-eclampsia (OR 0.72, 95% CI 0.62 to 0.85).^47^ Furthermore, a prospective study of over 30 000 nulliparous women found that intakes of fresh and dried fruits reduced the risk of pre-eclampsia.^48^ Consuming ≥330 g/day of fresh fruits was associated with an OR of 0.79 (95% CI 0.67 to 0.93), while consuming ≥4 g/day of dried fruits was associated with an OR of 0.79 (95% CI 0.68 to 0.92). Moreover, a 2015 prospective unmatched case–control study found that in a cohort of 453 pregnant women, the adjusted OR (AOR) of consuming fruits or vegetables no less than three times a week significantly reduced the risk of pre-eclampsia: fruits 0.51 (95% CI 0.29 to 0.91); vegetables 0.46 (95% CI 0.24 to 0.90).^49^ Later research in 2019 reported that higher vegetable intake, though not fruit intake, was associated with a lower risk of pre-eclampsia; women in the highest quartile of vegetable intake had an adjusted RR of 0.44 (95% CI 0.04 to 0.98) when compared with those in the lowest quartile.^50^ The same study also found that those in the highest quartile of vegetable intake had an adjusted RR of 0.44 (95% CI 0.24 to 0.8) for proteinuria versus those in the lowest quartile.^50^

#### Diets rich in ω-3 long-chain fatty acids

A prospective cohort study in Massachusetts found that women who consumed 100 mg/day of the ω-3 LC-PUFAs, docosahexaenoic acid (DHA) along with eicosapentaenoic acid (EPA), had a non-significant reduction in developing pre-eclampsia: OR 0.84 (95% CI 0.69 to 1.03).^46^ The same study found that intake of one portion of fish per day was associated with a non-significant reduction: OR 0.91 (95% CI 0.75 to 1.09). Later research in Denmark reported that EPA+DHA intakes ≥250 mg/day were associated with reduced risk of severe pre-eclampsia: RR 0.77 (95% CI 0.60 to 0.99), but not total pre-eclampsia,^53^ as highlighted previously in this review.^54^ However, fish, unlike ω-3 LC-PUFA supplements, contains bioactive peptides with antihypertensive, anti-inflammatory and antioxidant activities.^55^ Therefore, ideally EPA and DHA should be obtained from fish sources; consuming 8 ounces (227 g) of mixed seafood per week could provide~250 mg DHA+EPA per day.^56^

#### High-fat, sugar and salt-rich diets

The MoBa found that a diet characterised by higher intakes of processed meat, salty snacks and sweet drinks was associated with increased risk of pre-eclampsia (OR 1.21, 95% CI 1.03 to 1.42).^47^ The same study found that consumption of sugar-sweetened beverages was linked to a significantly increased risk of pre-eclampsia; an intake of ≥125 mL/day was found to be associated with an OR of 1.27 (95% CI 1.05 to 1.54).^48^ Moreover, the OR for consuming ≥1000 mL/day of carbonated sugar-sweetened beverages was 2.04 (95% CI 1.21 to 3.45).

#### Western dietary patterns

Research has demonstrated that women with greater adherence to a western diet are at increased risk of pre-eclampsia.^51 52 57^ In an Iranian case–control study of 510 pregnant women, a diet characterised by a high intake of red meat, processed meat, fried potatoes and pickles increased the risk of pre-eclampsia nearly sixfold (OR 5.99, 95% CI 3.41 to 10.53).^52^ A prospective cohort study in over 55 000 Danish women defined the western diet as characterised by high consumption of potatoes, meat, margarine and white bread; the OR for pre-eclampsia for women adhering to this diet was 1.40 (95% CI 1.11 to 1.76).^57^ The same study also found that adhering to a ‘seafood diet’, characterised by high fish and vegetable intake, significantly lowered the risk of pre-eclampsia: OR 0.79 (95% CI 0.65 to 0.97).^57^

#### New Nordic dietary pattern

Adherence to the New Nordic Diet (NND) in over 72 000 women from the MoBa study was assessed.^58^ High adherence to the NND was defined by various parameters such as eating ≥24 meals per week, eating cabbage at least two times per week and drinking at least six times as much water as sugar-sweetened beverages. In multivariate adjusted models, higher versus lower adherence to the NND was associated with reduced risk of total pre-eclampsia (OR 0.86, 95% CI 0.78 to 0.95) and early pre-eclampsia (OR 0.71, 95% CI 0.52 to 0.96).^58^

#### ‘Dietary Approaches to Stop Hypertension’ dietary pattern

A recent Chinese case–control study suggested that adherence to a ‘Dietary Approaches to Stop Hypertension (DASH)’-style diet reduced the risk of pre-eclampsia.^59^ Participants in the fourth quartile of the DASH score had an OR of 0.53 (95% CI 0.36 to 0.78) when compared with those in the lowest quartile.^59^ The DASH diet is rich in fruits, vegetables, whole grains, low-fat dairy and plant protein but low in red meat, processed meat, sweets and sugar-sweetened beverages.^60^ Conversely, research in the Danish National Birth Cohort found that greater adherence to the DASH diet did not significantly reduce pre-eclampsia risk.^61^ However, when looking at sodium intake in isolation, women who had a median sodium intake of 3.7 g/day versus those with a median sodium intake of 2.6 g/day had a 20% greater risk of developing pre-eclampsia.^61^

#### Mediterranean-style diet

In an observational study of reproductive Australian women, the association of pre-pregnancy dietary patterns and the risk of developing hypertensive disorders of pregnancy was examined.^62^ Women who had a ‘low adherence’ to a Mediterranean-style dietary pattern were found to be at increased risk of hypertensive disorders of pregnancy compared with those with high adherence (OR 1.41, 95% CI 1.18 to 1.56).^62^

In the ESTEEM (Effect of Simple, Targeted Diet in Pregnant Women With Metabolic Risk Factors on Pregnancy Outcomes) study, intercity pregnant women in five UK maternity units with metabolic risk factors were randomised to a Mediterranean-style diet (593 women) versus usual care (612 women).^63^ Women in the intervention arm consumed more nuts (70.1% vs 22.9%) and extra virgin olive oil (93.2% vs 49.0%) than controls; increased their intake of fish (p<0.001), white meat (p<0.001) and pulses (p=0.05); and reduced their intake of red meat (p<0.001), butter, margarine and cream (p<0.001). However, there was no significant reduction in the composite maternal outcomes, including pre-eclampsia; there was a relatively low incidence of pre-eclampsia as 73% of the participants were multigravida.^63^

#### Recommendations

As diet is a modifiable risk factor, a healthy, balanced diet should help reduce the risk of pre-eclampsia.^56^ WHO recommends an intake of ≥400 g of fruits and vegetables per day for general health.^64^ Pregnant women should eat a diet rich in fruits and vegetables, nuts, whole grains, legumes, olive oil and rich in fish; 8 ounces (227 g) of mixed seafood per week with selected types of fish can lead to a consumption of ≥250 mg DHA+EPA per day.^56^ Limiting the intake of foods high in fat, salt and sugar, including sugar-sweetened beverages, and reducing the intake of red and processed meat may also help reduce the risk.

### Vitamin D

There is some controversy about the amount of vitamin D that is necessary for avoidance of deficiency and the required concentration of the serum vitamin D metabolite, 25(OH)D, for adequacy. For women/pregnant women, an adequate intake (Recommended Dietary Allowance/Adequate Intake) of vitamin D is often defined as 15 µg (600 IU)/day, with a serum concentration of <50 nmol/L (>20 ng/mL) of 25(OH)D representing deficiency.^65 66^

#### Mechanisms by which vitamin D may protect against pre-eclampsia

Vitamin D may be protective by its ability to modulate proinflammatory responses, promote angiogenesis, decrease BP and maintain trophoblast survival capacity and immunological tolerance.^67–71^ Moreover, 25(OH)D deficiency is associated with endothelial dysfunction whereas replacement of vitamin D results in decreased oxidative stress.^72^

#### Observational studies of vitamin D in pre-eclampsia

The interest in vitamin D status in recent years means that a plethora of studies have looked at the effect of vitamin D on pre-eclampsia risk. A number of systematic reviews have shown an association between vitamin D status and pre-eclampsia, with some showing that serum vitamin D deficiency, that is, 25(OH)D <50 nmol/L, was a cut-off for increased risk while others found the cut-off to be vitamin D insufficiency, that is, <75 nmol/L.^73–80^ These studies suggest that treatment of vitamin D deficiency is necessary before pregnancy.

#### RCTs of vitamin D in pre-eclampsia

Evidence from a 2015 systematic review of 13 RCTs showed that vitamin D supplementation in pregnancy was associated with increased circulating 25(OH)D concentration but did not prevent pre-eclampsia.^81^ Similarly, a 2017 systematic review and meta-analysis of 27 RCTs with 28 000 women showed a non-significant reduction in vitamin D on pre-eclampsia risk.^82^ A 2019 Cochrane update investigated 30 trials that assessed 7033 women.^83^ A total of 22 trials involving 3725 pregnant women looked at supplementation with vitamin D alone versus placebo/no intervention; the risk of pre-eclampsia appeared to be reduced (RR 0.48, 95% CI 0.30 to 0.79); four trials of 499 women had moderate certainty evidence.^83^ Supplementation with vitamin D and calcium versus placebo/no intervention involved nine trials with 1916 pregnant women; the risk of pre-eclampsia was probably reduced (RR 0.50, 95% CI 0.32 to 0.78); four trials with 1174 women had moderate certainty evidence.^83^ A 2020 systematic review and meta-analysis of 27 RCTs included 4777 participants of whom 2487 were in the vitamin D-treated arm and 2290 were in the control arm.^84^ Vitamin D supplementation was associated with a reduced risk of pre-eclampsia (OR 0.37, 95% CI 0.26 to 0.52, I^2^=0%).^84^ A further systematic review and meta-analysis from 2020 incorporated 55 studies.^80^ Fixed-effects meta-analysis of the RCTs indicated that vitamin D supplementation was a prevention factor for pre-eclampsia; however, random-effects meta-analysis of trials found no significant association between vitamin D and pre-eclampsia.^80^ The most recent summary (2021) described itself as an ‘umbrella analysis’ and found a reduced risk of developing pre-eclampsia among pregnant women who received vitamin D supplementation (RR 0.62, 95% CI 0.43 to 0.91, I^2^=0%, 12 studies, n=1353).^85^

When considering the evidence outlined, multiple studies suggest that the risk of pre-eclampsia was *probably* reduced with vitamin D supplementation. Many investigators noted that well-designed clinical trials with vitamin D supplementation are still needed. However, observational studies suggest that it is important to reduce the occurrence of vitamin D deficiency/insufficiency in pregnancy.

#### Recommendation

Vitamin D supplementation in pregnant women is frequently required to achieve sufficient status as recommended by vitamin D guidelines so pregnant women should take a daily vitamin D supplement of 10–25 µg (400–1000 IU) to ensure they are not deficient. This may reduce their risk of pre-eclampsia. Considering the significant variation in individual vitamin D status and response to supplementation, screening may prove beneficial in defining optimal dosage.^86 87^

### Calcium

#### Potential protective mechanism of calcium in pre-eclampsia

Calcium intake may regulate BP by increasing intracellular calcium concentration.^88^ Low calcium intake can lead to high BP by stimulating the release of renin and parathyroid hormone.^88^ The release of these hormones increases intracellular calcium concentration in smooth muscle cells, causing vasoconstriction, increased peripheral vascular resistance and heightened BP.^88^ Calcium supplementation affects uteroplacental blood flow by reducing arterial vasoconstriction in the uterine and umbilical arteries.^89^ Furthermore, some research suggests that calcium supplementation may have an anti-inflammatory effect and may also prevent endothelial cell activation as seen in pre-eclampsia.^90^

Low calcium intake decreases plasma calcium concentration leading to activation of the renin-angiotensin-aldosterone system (RAAS). Activation of the RAAS stimulates the release of parathyroid hormone and parathyroid hypertensive factor and the release of calcitriol. This results in increased calcium concentration in the vascular smooth muscle cell causing vasoconstriction and results in high BP.^88^

#### Evidence for calcium in reducing pre-eclampsia risk

A relationship between low calcium intake and heightened risk of pre-eclampsia was first suggested in 1980.^91^ Systematic reviews and RCTs of calcium supplementation in pregnancy provide further evidence for this link.^82 92–96^ The protective effect of calcium supplementation on pre-eclampsia is well established with high-dose calcium supplementation (≥1 g/day); there is less research available on low-dose supplementation (<1 g/day).^94^ Daily 500 mg calcium supplementation for more than 6 months of pregnancy has been shown to reduce risk of pre-eclampsia by 45% (RR 0.55, 95% CI 0.33 to 0.93).^97^ However, supplementation with 500 mg calcium per day before 20 weeks’ gestation does not significantly reduce the risk of pre-eclampsia in women who had previous pre-eclampsia or eclampsia.^97^ Calcium supplementation appears particularly beneficial in women with a low calcium intake and women at heightened risk of pre-eclampsia.^82 93 94^ It is of interest that a 500 mg calcium supplement in non-pregnant women who had had previous pre-eclampsia significantly reduced systolic and mean arterial pressure.^98^ Although most guidelines recommend calcium supplementation for the prevention of pre-eclampsia,^99^ according to the WHO, there is currently insufficient evidence to suggest the point at which it should be initiated.^100^

Meertens and colleagues used a decision analytical model to assess the cost-benefit of 1 g/day calcium supplementation from 20 weeks’ gestation to delivery.^101^ They determined that supplementing all pregnant women, as opposed to only those at heightened risk of pre-eclampsia or those with low dietary calcium intake, would have a greater impact on reducing incidence of pre-eclampsia and would be more cost-effective.^101^

#### Recommendation

The evidence suggests that increasing calcium intake in pregnancy may effectively reduce pre-eclampsia incidence, especially among populations at heightened risk. All pregnant women should be supplemented with 1 g calcium per day from 20 weeks’ gestation to delivery. Women at heightened risk of pre-eclampsia and/or with a low dietary calcium intake should take a calcium supplement of 1–2 g/day during pregnancy to reduce the risk.^100^

### Selenium

The biological actions of the trace element, selenium, are mainly mediated by selenoproteins encoded by human genes that contain the amino acid, selenocysteine.^102–104^ There is an extremely wide range of intake of selenium seen across the world.^105 106^ Intake is quite high in North America but much lower in Europe.^103 107 108^

#### How does selenium affect the risk of pre-eclampsia?

Through their multiple effects, selenoproteins can reduce oxidative stress, endoplasmic reticulum stress and inflammation which are characteristics of pre-eclampsia.^109 110^ Further details of the mechanisms can be found in Rayman *et al*.^111 112^ Genetic evidence from a large Norwegian cohort showed that women with pre-eclampsia were significantly more likely to carry the A allele of the *selenoprotein S* G105A promoter polymorphism; this allele is less likely to degrade misfolded proteins from the ER, a main role of selenoprotein S, resulting in an increase in inflammatory cytokines.^113–115^

#### Epidemiology of selenium in pre-eclampsia

A systematic review and meta-analysis was published in 2016 that looked at 13 studies with 1515 participants.^116^ It showed an inverse association of blood selenium level with the risk of pre-eclampsia and that supplementation with selenium significantly reduces the incidence of pre-eclampsia. However, this study has been criticised for its methodology.^117^ A later study (2021) nicely summarised the associations between selenium status or selenium supplementation and pregnancy-induced hypertensive disorders.^118^ Specific issues affecting pre-eclampsia are discussed below.

#### Observational studies

**Figure 3:**
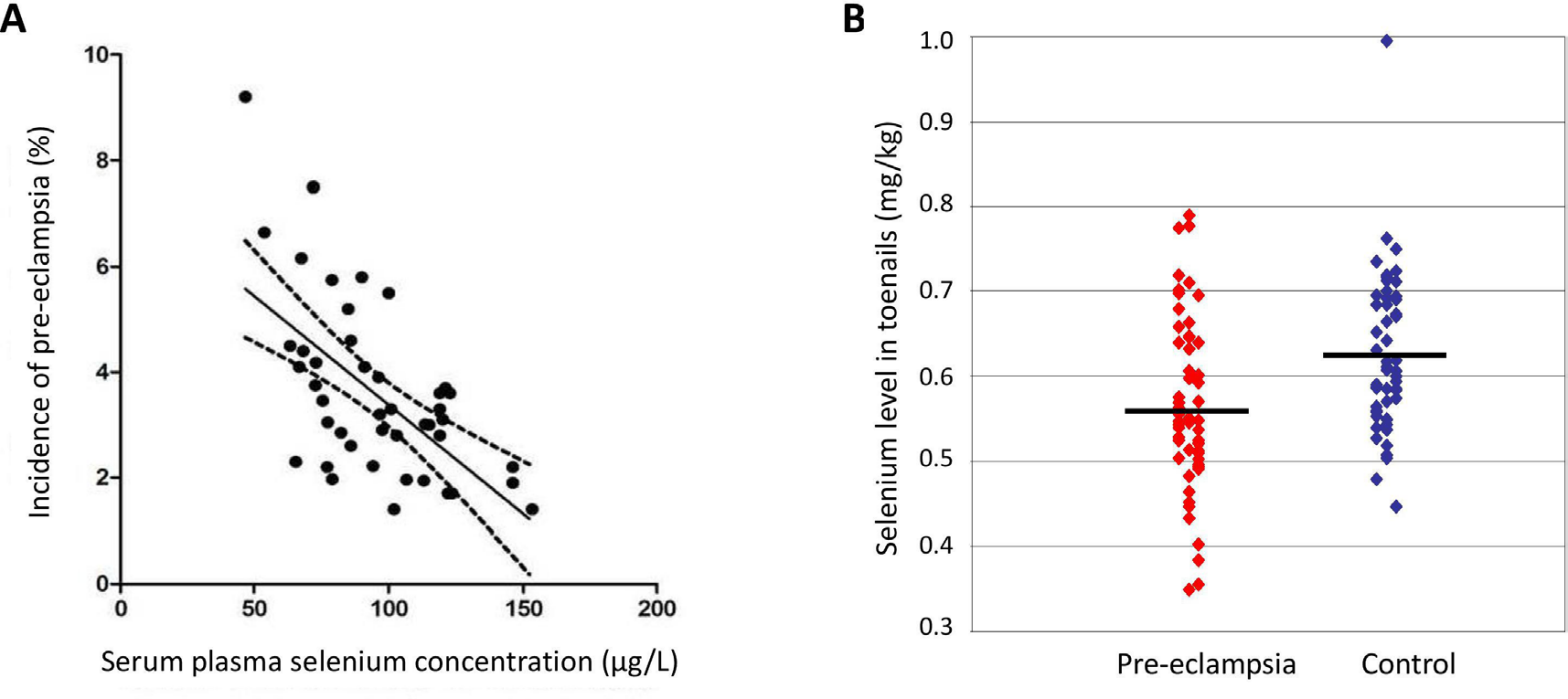
(A) Correlation between incidence of pre-eclampsia in various countries and serum/plasma selenium concentration (adapted from Vanderlelie and Perkins [119]). (B) Distribution of toenail selenium concentrations in pre-eclamptic and control subjects. *Horizontal bars*, median values (adapted from Rayman *et al* 127).

An epidemiological study of 45 countries found that a plasma selenium concentration of ≥95 µg/L correlated with a lower incidence of pre-eclampsia (see figure 3A).^119^ Significantly lower levels of selenoenzymes such as glutathione peroxidase (GPX) and thioredoxin reductase have been found in serum, plasma and placenta samples from pre-eclamptic women than in those from matched healthy controls.^120–126^

In a case–control study of 53 Oxford women of low selenium status, the concentration of selenium in toenail cuttings (laid down from 3 to 12 months previously to include pre-pregnancy) of women with pre-eclampsia was significantly lower than that of matched controls (see figure 3B).^127^ Women in the bottom tertile of toenail selenium were 4.4 times more likely to have pre-eclampsia.^127^

In a study from the Boston Birth Cohort, 1274 women were followed and levels of selenium and other trace minerals from red blood cells collected within 24–72 hours after delivery were measured.^128^ No association was observed between selenium and pre-eclampsia in that cohort, perhaps because American populations are selenium replete.^103 107 108^ However, the large MoBa study in Norway had a similar finding^118^; maternal selenium concentrations were measured in whole blood collected around gestational week 18 in a subset of 2572 pregnant women. No significant associations were found between whole-blood selenium concentration (median 102 μg/L (IQR 89–117)) and pre-eclampsia.^118^ Similarly, there was no relationship between reported selenium intake from diet (measured in 69 972 women at 22 weeks) or dietary supplements (total median intake 53 (IQR 44–62) µg/day) and pre-eclampsia.^118^

#### RCTs of selenium in pregnancy

In a double-blinded, placebo-controlled pilot trial, 230 primiparous pregnant women in Oxford, of relatively low selenium status (median whole-blood selenium 103 (range: 66.3–261.4) µg/L), were randomised to selenium (60 µg/day, as selenium-enriched yeast) or placebo treatment from 12 to 14 weeks of gestation until delivery.^111 112^ Whole-blood selenium concentration, toenail selenium concentration, plasma selenoprotein P (a recognised marker of selenium status^129^) and plasma GPX activity were measured at various gestational ages.^111 112^ The primary outcome measure was serum soluble vascular endothelial growth factor receptor-1 (sFlt-1), an antiangiogenic factor linked to the risk of pre-eclampsia.^111^ The concentration of sFlt-1 was significantly lower at 35 weeks in the selenium-treated group than in the placebo group in participants in the lowest quartile of selenium status at baseline (mean 0.70, 95% CI 0.49 to 0.98, p=0.039) showing that supplementation affected the risk of pre-eclampsia in those of low selenium status. In a later study, the level of GPX and selenoprotein P in those women suggested a requirement for a higher rate of supplementation than 60 µg/day, or longer treatment.^111 112 130 131^

Selenium status was measured in toenail clippings at 16 weeks’ gestation; this is a measure of pre-pregnancy selenium status as the toenails would have been laid down before pregnancy. Toenail selenium was significantly correlated with baseline whole-blood selenium concentration and diastolic BP.^112^ When we combined the outcomes of pre-eclampsia and pregnancy-induced hypertension (PIH),^132^ the median toenail selenium concentration in women who developed pre-eclampsia/PIH was significantly lower than that in other women^112^ as in the earlier study.^127^ Among the selenium-related risk factors, only toenail selenium significantly affected the OR for pre-eclampsia/PIH suggesting that pre-pregnancy status has a more significant effect on risk than status from week 12.^112^

The disparity in results from the large MoBa cohort study and the UK studies is hard to reconcile. Selenium status at 18 weeks in the MoBa cohort and at 12 weeks in the UK trial was the same; as selenium status falls with gestational week,^111^ the MoBa cohort may have had a somewhat higher status than that in the UK. The median selenium intake in MoBa was 53 µg/day which is close to the recommended intake for pregnant women of 60 μg/day.^133 134^ Intake in the UK was not measured but 2008/2010 data from the UK National Diet and Nutrition rolling programme give a median of 39 µg/day in women of 19–64 years^135^ which is considerably lower than that in MoBa. Other differences between the two groups may be the consumption of relatively high amounts of fish and seafood in the MoBa cohort which contributed 23% of the total selenium intake and are known to contain a different form of selenium (selenoneine).^118 136^

There are indications from the UK studies that periconceptional selenium status may be more important than status after 12 weeks.^112 127^ This would fit with the recognised oxidant challenge at the initiation of intervillous blood flow around pregnancy weeks 8–10^137^ and suggest that it is important to have adequate selenium status at a very early stage of pregnancy.

#### Recommendation

Low selenium status may be a risk for pre-eclampsia in women with low selenium status though the level of status at which the risk increases is unclear. Women in North America and other parts of the world where selenium status is adequate do not need to worry,^106^ but women with low selenium status, for example, countries such as the UK with relatively low status,^106^ should try to increase their intake of selenium-containing foods, ideally by consuming more than two fish/seafood portions per week.^112^ Alternatively, women could take a pregnancy supplement containing selenium (say, 50 - 100 μg/d) as soon as they know they are pregnant and preferably when planning pregnancy.

### Folic acid, vitamin B_12_ and multivitamins/minerals

Some 85% of studies showed that women with pre-eclampsia had a higher serum homocysteine concentration than those without pre-eclampsia.^138^ A high concentration of homocysteine could be associated with a lower level of folate or vitamin B_12_.^139^ Folate requirements are increased in pregnancy due to the rapid division of cells in the fetus and increased urinary losses.^140^ Sufficient dietary intake of folate (supplemented as folic acid) during pregnancy is important for normal placentation, the growth and development of the fetus, and to prevent neural tube defects.^141^ Folate may play a protective role in pre-eclampsia as it is involved in mechanisms that lower BP, reduce oxidative stress and restore endothelial function.^142 143^

A recent meta-analysis of 19 studies looked at the serum vitamin B_12_ concentrations of women with pre-eclampsia and found that they were significantly lower than those of healthy pregnant women (mean −15.24 pg/mL, 95% CI −27.52 to −2.954, p<0.015).^138^ However, the heterogeneity between the studies was very high (I^2^=97.8%; p=0.0103) casting doubts on the validity of the observation. Since that meta-analysis, two additional studies found no association between vitamin B_12_ concentrations in maternal blood and pre-eclampsia.^144 145^ However, both studies also showed that higher homocysteine concentration and lower folate level during early pregnancy were associated with pre-eclampsia.^144 145^

Multivitamins/minerals, which generally contain folic acid and vitamin B_12_, may also reduce the risk of pre-eclampsia through the protective mechanisms of the individual nutrients, some of which may work in synergy.^146^

#### Evidence for an effect of folic acid/multivitamin/minerals in pre-eclampsia

Some studies suggest that maternal folic acid supplementation may reduce the risk of pre-eclampsia.^147–149^ A 2009 longitudinal study in Denmark examined the relationship between periconceptional multivitamin/mineral use and pre-eclampsia; there were 668 cases of pre-eclampsia (2.3%), and 18 551 women (65%) reported periconceptional multivitamin use. Regular users with a BMI of 22 kg/m^2^ had a 20% reduced risk of pre-eclampsia compared with non-users.^150^ Multivitamin/mineral use after conception was only associated with a reduced risk of pre-eclampsia in women with a BMI <25 kg/m^2^.^150^

A 2016 prospective cohort study in Australia found that women who took a multivitamin/mineral preparation during the first trimester of pregnancy had a 67% reduced risk of developing pre-eclampsia.^147^ Stratifying women by BMI revealed that this effect was not significant in those with a BMI <25 kg/m^2^ while the AOR in the overweight and obese cohort did reach significance.^147^ These findings suggest that multivitamin/mineral use in the first trimester may be of importance, especially for those with a BMI ≥25 kg/m^2^, contradicting the findings of the previous study.^150^ Moreover, a 2020 cohort study concluded that periconceptual and early pregnancy multivitamin/mineral use was significantly associated with reduced risk of pre-eclampsia in overweight women but not in underweight, normal-weight or obese women.^151^

A 2018 meta-analysis examined four studies in relation to folic acid supplementation and pre-eclampsia risk.^145^ Although the pooled results determined that folic acid supplementation is related to reduced risk of pre-eclampsia (RR 0.69, 95% CI 0.58 to 0.83), the heterogeneity was high across the studies examined (I^2^=86%); furthermore, subgroup analysis revealed that the use of multivitamins containing folic acid reduces the risk of pre-eclampsia while folic acid alone had no significant effect.^145^ An earlier meta-analysis also revealed that taking a periconceptual multivitamin or multivitamin/mineral supplement containing folic acid significantly reduced the risk of pre-eclampsia (RR 0.67, 95% CI 0.51 to 0.89), but that folic acid supplementation alone did not.^152 153^ In a 2021 meta-analysis, 20 studies with 359 041 patients were identified including three RCTs and 17 cohort studies. Pooled estimates showed an RR of 0.83 (95% CI 0.74 to 0.93, p=0.0008) for association between low-dose folic acid and the risk of pre-eclampsia.^154^ Multivitamins/minerals were not assessed.

A clinical trial run in five countries randomised 2464 pregnant women with at least one high-risk factor for pre-eclampsia to high-dose supplementation (4.0 mg folic acid per day) from 8 to 16 weeks of gestation until delivery.^155^ Pre-eclampsia occurred in 14.8% women in the folic acid group and 13.5% in the placebo group (RR 1.10, 95% CI 0.90 to 1.34, p=0.37) showing no benefit of high-dose folic acid.^155^

#### Recommendation

To reduce the risk of pre-eclampsia, women planning to become pregnant should take a daily multivitamin/mineral supplement containing folic acid (400 μg) and vitamin D (≥10 µg), and in countries with low selenium status, selenium (say, 50 μg). If pregnancy is unplanned, such a supplement should start as soon as possible in pregnancy; it should be taken for at least the first trimester.

## Conclusion

The dietary factors we have described as being implicated in reducing the risk of pre-eclampsia are summarised in table 4. However, note that dietary recommendations should be considered in combination with other preventive actions such as a screening policy or pharmacological agents that may be appropriate for high-risk groups.

**Table 4:** Factors implicated in reducing the risk of pre-eclampsia (Recommended Dietary Allowance (RDA) included in column 1)

Factor	Recommendation	Further relevant advice
Maternal weight	Excessive weight gain during pregnancy and between pregnancies should be avoided. Women should ideally be of a healthy body weight (BMI) prior to conception. Recommendations are that underweight women (BMI ≤18.5) should put on between 13 and 18 kg; normal-weight women (BMI 18.5–24.9) should put on between 11.5 and 16 kg; overweight women (BMI 25–29.9) should put on between 7 and 11.5 kg and obese women (BMI ≥30) should put on no more than 5–9 kg.	A woman aiming to reduce her BMI prior to pregnancy should do so safely preferably with the help of a suitable healthcare professional.
Fibre	A high-fibre diet is recommended for pregnant women and those at risk of pre-eclampsia. Women should aim for a fibre intake of 25–30 g/day to reduce the risk of pre-eclampsia.	Higher fibre intake can reduce blood cholesterol, blood pressure and inflammation and may also aid in weight management.
Prebiotics and probiotics	Consume milk-based probiotics where possible as part of a normal diet.	Further research is required to determine the quantity, timing and efficacy of probiotics to reduce pre-eclampsia risk.
Dietary patterns	Pregnant women should aim to consume ≥400 g of fruits and vegetables per day and ≥250 mg/day of docosahexaenoic/eicosapentaenoic acid by consuming ~230 g (8 ounces) of mixed seafood per week. High fat, salt, sugar foods and red and processed meats should be limited.	Avoid raw fish and fish with a high mercury content (shark, swordfish, king mackerel, tilefish, marlin, orange roughy, bigeye tuna) during pregnancy.
Vitamin D RDA 15 μg/day	Daily vitamin D supplement of 10–25 µg (400–1000 IU); stay well away from the upper limit of 100 μg (4000 IU).	A woman’s current vitamin D status can be measured and may be helpful in defining optimum dosage.
Calcium RDA 1000 mg/day	All pregnant women to be supplemented with 1 g calcium per day from 20 weeks’ gestation to delivery. Women at heightened risk of pre-eclampsia and/or with a low dietary calcium intake should take a calcium supplement of 1–2 g/day during pregnancy.	Calcium supplement, for example, carbonate or citrate. Women are likely to have low dietary calcium intake if they do not consume dairy products which are a major calcium source.
Selenium RDA 60 μg/day	Women who are likely to have low selenium status (eg, in the UK), should increase their intake of selenium-rich foods, such as fish/shellfish. Alternatively, they should take a multivitamin/multimineral containing selenium as soon as they know they are pregnant and preferably when planning pregnancy.	Brazil nuts are a good source of selenium but the dose can be very high, so it is a risky way of supplementing selenium. Keep intake down to four per week if taking regularly.
Multivitamins/multiminerals	Women should take a multivitamin/multimineral supplement containing folic acid, vitamin D (unless taking separately) and selenium (if status is low) if they are planning on becoming pregnant. If pregnancy is unplanned, the woman should start the supplement as soon as possible. The supplement should be taken for at least the first trimester.	This supplement may be of particular importance for those who are overweight. Iodine is important for fetal brain development and should be included for a woman who does not eat dairy products or fish in a country that does not have iodised salt. The usual dose in pregnancy is 150 μg/day, usually as potassium iodide. Avoid taking a kelp supplement.

BMI, body mass index.

## Data Availability

All data are already published; no additional data are available. Data from our studies on selenium are also published.
