# Supplementary material for: Dietary factors that affect the risk of pre-eclampsia
Source: BMJ Nutr Prev Health. 2022 Jun 6;5(1):118–33. doi: 10.1136/bmjnph-2021-000399 (PMC9237898; doi:10.1136/bmjnph-2021-000399)
Supplement: Supplementary data [file bmjnph-2021-000399supp001.pdf]

Supplementary Information

Dietary factors that affect the risk of pre-eclampsia

Abigail Perry BSc, RD; Anna Stephanou BSc, RD; Margaret P Rayman DPhil (Oxon), RNutr

Department of Nutritional Sciences, Guildford, United Kingdom

Foods and nutrients with low or no evidence of benefit

Antioxidants: vitamin C and vitamin E

As oxidative stress plays a fundamental role in the pathophysiology of preeclampsia,[1] it was previously believed that supplementation with antioxidants such as vitamins C and E might have a protective role. A randomised control trial (RCT) conducted in 1999 found that supplementation with vitamins C and E was associated with a significant reduction in plasma markers of endothelial and placental dysfunction.[2] However, five other studies, including a 2008 Cochrane review of ten trials, revealed that supplementation with vitamins C and E did not significantly reduce the risk of preeclampsia.[3-7] Nonetheless, a small clinical trial in one centre in Malaysia has shown some potential benefit of palm oil vitamin E in the form of a tocotrienol-rich fraction (TRF, 100 mg daily) which is a more potent antioxidant and has superior anti-inflammatory properties than tocopherol in preventing preeclampsia.[8]

ω-3 Long-chain polyunsaturated fatty acids (ω-3 LC-PUFAs)

As ω-3 LC-PUFAs are implicated in anti-inflammatory and anti-oxidative pathways, ω-3 LC-PUFA supplementation has been queried as a potential therapeutic intervention in preeclampsia.[9] However, the six studies shown in Table 2 show that ω-3 LC-PUFA supplementation in pregnancy does not significantly reduce the risk.[10-16]

Low-salt diet

High dietary sodium intake raises blood pressure therefore, women had previously been advised that lowering their salt intake might reduce their risk of preeclampsia.[17] However, research now shows that reducing the intake of salt has no effect on the risk of preeclampsia.[18]

Magnesium

Magnesium has a significant physiological role in the regulation of blood pressure and has been successfully implemented as a treatment for eclamptic seizures.[19] These findings led to the hypothesis that magnesium might be deficient in women with preeclampsia.[20] However, a 2014 Cochrane review of ten trials of magnesium supplementation involving 9090 women and their babies and a subsequent randomised trial of magnesium supplementation in 318 pregnant women suggested no effect on the risk of preeclampsia.[21,22]

Zinc

Some research suggests that low maternal zinc status may be associated with increased risk of preeclampsia;[23,24] however, there is no evidence of benefit for supplementation with 15 mg/day zinc sulphate in reducing preeclampsia risk.[25]

References

1. Redman CW, Sargent IL. Placental stress and pre-eclampsia: a revised view. *Placenta* 2009; **30 Suppl A**: S38-42.
2. Chappell LC, Seed PT, Briley AL, et al. Effect of antioxidants on the occurrence of pre-eclampsia in women at increased risk: a randomised trial. *Lancet* 1999; **354**(9181): 810-6.
3. Rumbold A, Duley L, Crowther CA, Haslam RR. Antioxidants for preventing pre-eclampsia. *Cochrane Database Syst Rev* 2008; **2008**(1): Cd004227.
4. Poston L, Briley AL, Seed PT, Kelly FJ, Shennan AH. Vitamin C and vitamin E in pregnant women at risk for pre-eclampsia (VIP trial): randomised placebo-controlled trial. *Lancet* 2006; **367**(9517): 1145-54.
5. Klemmensen A, Tabor A, Østerdal ML, et al. Intake of vitamin C and E in pregnancy and risk of pre-eclampsia: prospective study among 57 346 women. *Bjog* 2009; **116**(7): 964-74.
6. Basaran A, Basaran M, Topatan B. Combined vitamin C and E supplementation for the prevention of preeclampsia: a systematic review and meta-analysis. *Obstet Gynecol Surv* 2010; **65**(10): 653-67.
7. Salles AM, Galvao TF, Silva MT, Motta LC, Pereira MG. Antioxidants for preventing preeclampsia: a systematic review. *ScientificWorldJournal* 2012; **2012**: 243476.
8. Aminuddin NA, Sutan R, Mahdy ZA. Role of Palm Oil Vitamin E in Preventing Pre-eclampsia: A Secondary Analysis of a Randomized Clinical Trial Following ISSHP Reclassification. *Front Med (Lausanne)* 2020; **7**: 596405.
9. Jones ML, Mark PJ, Waddell BJ. Maternal dietary omega-3 fatty acids and placental function. *Reproduction* 2014; **147**(5): R143-52.
10. Middleton P, Gomersall JC, Gould JF, Shepherd E, Olsen SF, Makrides M. Omega-3 fatty acid addition during pregnancy. *Cochrane Database Syst Rev* 2018; **11**(11): Cd003402.
11. Williams MA, Zingheim RW, King IB, Zebelman AM. Omega-3 fatty acids in maternal erythrocytes and risk of preeclampsia. *Epidemiology* 1995; **6**(3): 232-7.

12. Oken E, Ning Y, Rifas-Shiman SL, Rich-Edwards JW, Olsen SF, Gillman MW. Diet during pregnancy and risk of preeclampsia or gestational hypertension. *Ann Epidemiol* 2007; **17**(9): 663-8.
13. Szajewska H, Horvath A, Koletzko B. Effect of n-3 long-chain polyunsaturated fatty acid supplementation of women with low-risk pregnancies on pregnancy outcomes and growth measures at birth: a meta-analysis of randomized controlled trials. *Am J Clin Nutr* 2006; **83**(6): 1337-44.
14. Makrides M, Duley L, Olsen SF. Marine oil, and other prostaglandin precursor, supplementation for pregnancy uncomplicated by pre-eclampsia or intrauterine growth restriction. *Cochrane Database Syst Rev* 2006; (3): Cd003402.
15. Horvath A, Koletzko B, Szajewska H. Effect of supplementation of women in high-risk pregnancies with long-chain polyunsaturated fatty acids on pregnancy outcomes and growth measures at birth: a meta-analysis of randomized controlled trials. *Br J Nutr* 2007; **98**(2): 253-9.
16. Imhoff-Kunsch B, Briggs V, Goldenberg T, Ramakrishnan U. Effect of n-3 long-chain polyunsaturated fatty acid intake during pregnancy on maternal, infant, and child health outcomes: a systematic review. *Paediatr Perinat Epidemiol* 2012; **26 Suppl 1**: 91-107.
17. Huang L, Trieu K, Yoshimura S, et al. Effect of dose and duration of reduction in dietary sodium on blood pressure levels: systematic review and meta-analysis of randomised trials. *Bmj* 2020; **368**: m315.
18. Duley L, Henderson-Smart D, Meher S. Altered dietary salt for preventing pre-eclampsia, and its complications. *Cochrane Database Syst Rev* 2005; (4): Cd005548.
19. Altura BM, Altura BT. Magnesium ions and contraction of vascular smooth muscles: relationship to some vascular diseases. *Fed Proc* 1981; **40**(12): 2672-9.
20. Roberts JM, Balk JL, Bodnar LM, Belizán JM, Bergel E, Martinez A. Nutrient involvement in preeclampsia. *J Nutr* 2003; **133**(5 Suppl 2): 1684s-92s.
21. Makrides M, Crosby DD, Bain E, Crowther CA. Magnesium supplementation in pregnancy. *Cochrane Database Syst Rev* 2014; **2014**(4): Cd000937.
22. de Araújo CAL, de Sousa Oliveira L, de Gusmão IMB, Guimarães A, Ribeiro M, Alves JGB. Magnesium supplementation and preeclampsia in low-income pregnant women - a randomized double-blind clinical trial. *BMC Pregnancy Childbirth* 2020; **20**(1): 208.
23. Wilson RL, Grieger JA, Bianco-Miotto T, Roberts CT. Association between Maternal Zinc Status, Dietary Zinc Intake and Pregnancy Complications: A Systematic Review. *Nutrients* 2016; **8**(10).
24. Zhu Q, Zhang L, Chen X, Zhou J, Liu J, Chen J. Association between zinc level and the risk of preeclampsia: a meta-analysis. *Arch Gynecol Obstet* 2016; **293**(2): 377-82.
25. Zahiri Sorouri Z, Sadeghi H, Pourmarzi D. The effect of zinc supplementation on pregnancy outcome: a randomized controlled trial. *J Matern Fetal Neonatal Med* 2016; **29**(13): 2194-8.
